# Roles of the hydroxy group of tyrosine in crystal structures of *Sulfurisphaera tokodaii* *O* ^6^-methylguanine-DNA methyltransferase

**DOI:** 10.1107/S2053230X21011055

**Published:** 2021-11-11

**Authors:** Makiko Kikuchi, Takahiro Yamauchi, Yasuhito Iizuka, Masaru Tsunoda

**Affiliations:** a Graduate School of Science and Engnieering, Iryo Sosei University, Iwaki, Fukushima, Japan; b Graduate School of Life Science and Technology, Iryo Sosei University, Iwaki, Fukushima, Japan; c Department of Pharmacy, Fukushima Rosai Hospital, Iwaki, Fukushima, Japan; d Faculty of Pharmacy, Iryo Sosei University, Iwaki, Fukushima, Japan

**Keywords:** *O* ^6^-methylguanine-DNA methyltransferases, DNA repair, tyrosine, *Sulfurisphaera tokodaii*, hydroxy group

## Abstract

*O* ^6^-Methylguanine-DNA methyltransferase (MGMT) removes cytotoxic *O* ^6^-alkyl adducts on the guanine base and protects the cell from genomic damage induced by alkylating agents. Although there are reports of computational studies on the activity of the enzyme with mutations at tyrosine residues, no studies concerning the crystal structure of its mutants have been found. In this study, the function of Tyr91 was investigated in detail by comparing the crystal structures of mutants and their complexes with substrate analogs. In this study, tyrosine, a conserved amino acid near the active-site loop in the C-terminal domain of *Sulfurisphaera tokodaii* MGMT (StoMGMT), was mutated to phenylalanine to produce a Y91F mutant, and the cysteine which is responsible for receiving the methyl group in the active site was mutated to a serine to produce a C120S mutant. A Y91F/C120S double-mutant StoMGMT was also created. The function of tyrosine is discussed based on the crystal structure of Y91F mutant StoMGMT. The crystal structures of StoMGMT were determined at resolutions of 1.13–2.60 Å. They showed no structural changes except in the mutated part. No electron density for deoxyguanosine or methyl groups was observed in the structure of Y91F mutant crystals immersed in *O* ^6^-methyl-2′-deoxyguanosine, nor was the group oxidized in wild-type StoMGMT. Therefore, the hydroxy group of Tyr91 may prevent the oxidant from entering the active site. This suggests that tyrosine, which is highly conserved at the N-terminus of the helix–turn–helix motif across species, protects the active site of MGMTs, which are deactivated after repairing only one alkyl adduct. Overall, the results may provide a basis for understanding the molecular mechanisms by which high levels of conserved amino acids play a role in ensuring the integrity of suicide enzymes, in addition to promoting their activity.

## Abstract 2

Structural analyses of *O* ^6^-methylguanine-DNA methyltransferases (MGMTs) and their mutants suggest that the highly conserved tyrosine at the N-terminus of the helix–turn–helix motif may play a protective role in MGMTs by preventing oxidants from entering the active site.

## 1. Introduction

The genomes of all organisms are always at risk. Fortunately, cells can detect and react to any damage to genomic integrity through a variety of DNA-repair mechanisms, each with its own target and mechanism. One of the most important DNA-repair mechanisms involves the enzyme *O* ^6^-methylguanine-DNA methyltransferase (also known as MGMT, AGT or OGT; EC 2.1.1.63; Kaina *et al.*, 2007 ▸). Its primary function is to remove highly cytotoxic *O* ^6^-alkyl adducts on the guanine base (*O* ^6^-alkyl G), protecting the cell from adverse biological effects induced by alkylating agents (Jacinto & Esteller, 2007 ▸; Zhong *et al.*, 2010 ▸).

MGMT is highly conserved in all organisms ranging from bacteria to mammals. It translocates alkyl adducts via a direct damage-reversal pathway from *O* ^6^-alkyl G-based oxygen to cysteine residues in the catalytic pocket. This unconventional mechanism repairs DNA but irreversibly deactivates MGMT, which is described as ‘suicide’ (Pegg, 2011 ▸). After the introduction of the alkyl group, the alkylated MGMT protein appears to be rapidly ubiquitinated and is susceptible to degradation by the proteasome, rather than being reconstituted and demethylated to reactivate the enzyme (Daniels *et al.*, 2000 ▸). In other words, one MGMT molecule can repair only one alkyl adduct.

The *Sulfurisphaera tokodaii* MGMT enzyme (StoMGMT) exhibits a typical MGMT protein structure consisting of two domains: a highly conserved C-terminal domain (CTD) that surprisingly overlaps with all available MGMT structures and an N-terminal domain (NTD) which, in contrast, differs greatly among MGMTs. The CTD contains a DNA-binding helix–turn–helix (HTH) motif, which is followed by an Asn hinge. This hinge precedes the –(V/I)PCHRV(V/I)– amino-acid sequence, which contains a conserved catalytic cysteine and an active-site loop, and is involved in substrate specificity. The catalytic network consists of cysteine, water, histidine and glutamic acid, similar to the catalytic triad of serine proteases (Daniels & Tainer, 2000 ▸).

In addition to the active-site loop sequence, there are 5–6 conserved amino acids in MGMTs. Among them, the tyrosine residue located at the entrance to the active pocket is said to play a role in promoting the reaction by rotating the target base of damaged DNA through steric and electrostatic effects (Hu *et al.*, 2008 ▸). Although there are reports of computational studies on the activity of the enzyme with mutations at tyrosine residues (Thirumal Kumar *et al.*, 2019 ▸), no studies of the crystal structures of its mutants have been found. In this study, we mutated two conserved amino acids in StoMGMT. Firstly, we mutated Tyr91, which is conserved in the vicinity of the active-site loop in StoMGMT, to phenylalanine to produce the Y91F mutant. Secondly, we mutated the cysteine which is responsible for receiving the methyl group in the active site to serine (C120S mutant). In addition, we created a double mutant that included both mutations (Y91F/C120S double mutant). We decided to investigate the function of Tyr91 in detail by comparing the crystal structures of these mutants and their complexes with substrate analogs.

## 2. Materials and methods

### 2.1. Macromolecule production

The STK_RS05355 gene encoding the MGMT protein from *S. tokodaii* strain 7^T^ was amplified from the genomic DNA of the strain (NBRC 100140G; obtained from NBRC, NITE, Kisarazu, Japan) using polymerase chain reaction (PCR). The PCR fragment was digested with NdeI and BamHI and cloned into a pET-11a expression vector. The site-directed mutants Y91F, C120S and Y91F/C120S were generated using the QuikChange Site-Directed Mutagenesis Kit (Agilent Technologies Japan, Tokyo, Japan). Each clone was transformed into *Escherichia coli* Rosetta-gami (DE3) cells and plated on plates containing Luria–Bertani (LB) agar with ampicillin and chloramphenicol. A single colony from the LB agar plate was used to inoculate the primary culture in 15 ml LB medium supplemented with 15 µl ampicillin (50 mg ml^−1^) and 15 µl chloramphenicol (34 mg ml^−1^), which was allowed to grow overnight at 310 K. A secondary culture was set up by adding an inoculum from the primary culture to 1 l LB medium supplemented with ampicillin (50 mg ml^−1^) and chloramphenicol (34 mg ml^−1^) to a final concentration of 0.1%. The culture was incubated for 12 h at 310 K. The cells were harvested by centrifuging the culture at 8000*g* for 15 min at 277 K. The pellet was resuspended in double the volume of lysis buffer (20 m*M* Tris–HCl pH 8.0, 50 m*M* NaCl) and sonicated on ice using an ultrasonicator (UD-201; Tomy Seiko Co., Tokyo, Japan) seven times using a 30 s on/60 s off cycle. The proteins were purified by heating the supernatant to 343 K for 30 min, followed by centrifugation of the precipitated biomolecules at 8000*g* for 15 min. As analyzed by SDS–PAGE, this treatment precipitated most of the proteins of the *E. coli* expression host, while the thermostable proteins remained soluble. The supernatant was brought to 50% saturation using ammonium sulfate, clarified by centrifugation and precipitated with 70% saturated ammonium sulfate. The resuspended protein was dialyzed overnight against 1 l dialysis buffer [50 m*M* potassium phosphate pH 6.5, 50 m*M* potassium chloride, 0.1 m*M* ethylenediaminetetraacetic acid (EDTA)] at 277 K using a 10 kDa molecular-weight cutoff membrane. The protein sample was applied onto a 5 ml HiTrap SP HP column (Cytiva, Marlborough, Massachusetts, USA) pre-equilibrated in dialysis buffer. The loaded column was washed with dialysis buffer and eluted using an increasing linear gradient of elution buffer (50 m*M* potassium phosphate pH 6.5, 550 m*M* potassium chloride, 0.1 m*M* EDTA) over 30 column volumes. The eluted protein was desalted against dialysis buffer using Amicon Ultra centrifugal filter units (Merck KGaA, Darmstadt, Germany). The production of the macromolecule is summarized in Table 1 ▸.

### 2.2. Crystallization

Purified StoMGMT was concentrated to 10 mg ml^−1^. The crystallization conditions were screened using the hanging-drop vapor-diffusion method. The experiments were performed at 293 K using several commercial screens. Diffraction-quality crystals were obtained from Index screen conditions 56, 63 and 69 (Hampton Research, Aliso Viejo, California, USA). Prior to data collection, the crystals were soaked in reservoir solution with 1 m*M* *O* ^6^-methyl-2′-deoxy­guanosine (hereafter referred to as *O* ^6^-mdG; Berry & Associates, Dexter, Michigan, USA) for 0.5–2 h. Crystallization conditions are given in Table 2 ▸.

### 2.3. Data collection and processing

The crystals were cryoprotected by transferring them into the perfluoropolyether oil Fomblin Y (Merck KGaA, Darmstadt, Germany) and were flash-cooled in a cold stream of nitrogen gas at 100 K immediately before data collection. The diffraction data for StoMGMT were collected on the macromolecular crystallography beamlines BL-1A and BL-5A at the Photon Factory (PF), Tsukuba, Japan. Images were indexed and integrated using *XDS* (Kabsch, 2010*a* ▸,*b* ▸) followed by data reduction and scaling using *AIMLESS* (Evans & Murshudov, 2013 ▸). All calculations were carried out within the *CCP*4*i* interface (Potterton *et al.*, 2018 ▸) using the *CCP*4 software suite (Winn *et al.*, 2011 ▸). Data-collection statistics are summarized in Table 3 ▸.

### 2.4. Structure solution and refinement

*MOLREP* (Vagin & Teplyakov, 2010 ▸) was used for molecular replacement using the structure of wild-type StoMGMT (PDB entry 1wrj; M. Kuroda, M. Tsunoda & K. T. Nakamura, unpublished work) as a search model. The coordinates of the structure were refined using several cycles of *REFMAC*5 (Murshudov *et al.*, 2011 ▸) and *Coot* (Emsley *et al.*, 2010 ▸). Water and ligand molecules were added via *Coot* and modeled manually into the electron density. Structures were generally validated using several tools in *Coot* and the wwPDB validation service (Young *et al.*, 2017 ▸) during deposition. Evaluation of the Ramachandran plot using *MolProbity* (Chen *et al.*, 2010 ▸) showed that all residues were in the allowed regions (94.6–96.6% were in favored regions). All figures showing 3D protein structures were produced using the *PyMOL* molecular-visualization system (https://www.pymol.org/). The refinement statistics are summarized in Table 4 ▸.

## 3. Results and discussion

The crystal structures of StoMGMT were determined at resolutions of 1.13–2.60 Å. Almost all amino-acid residues of StoMGMT could be assigned in the final models, except for some residues at the C-terminus (Val150–Lys156).

**Figure 1:**
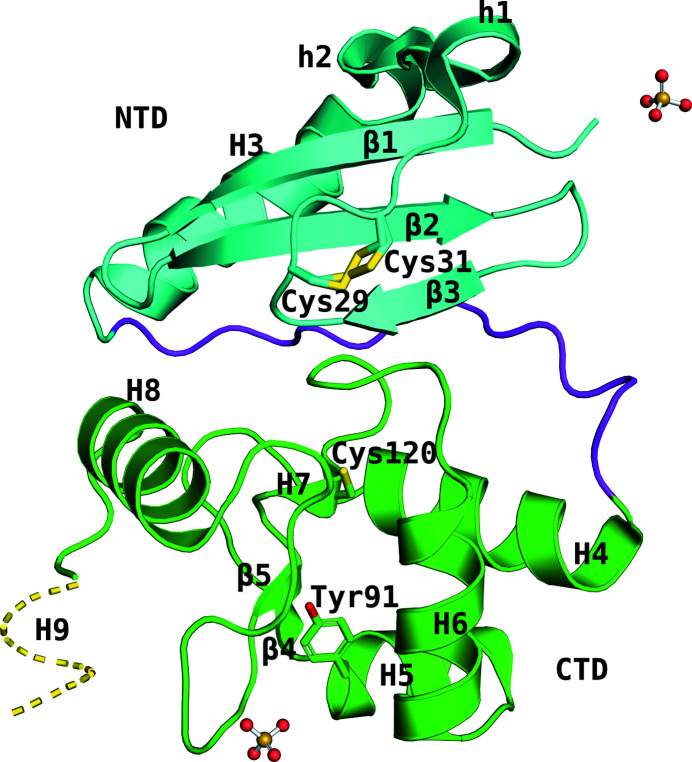
Overall structure of wild-type StoMGMT. The protein is shown as a ribbon diagram with the NTD (residues 1–55) colored cyan, the connecting loop colored purple and the CTD (residues 70–156) colored green. β indicates β-strand, while h and H indicate 3_10_-helix and α-helix, respectively. The Cys29–Cys31 disulfide bond is found in two different conformations. The sulfate ions are shown using a ball-and-stick model. H9 is ordered in the structures of the C120S and Y91F mutants.

StoMGMT shares a high percentage of similarity with MGMTs from human (43%; Daniels *et al.*, 2000 ▸; Wibley *et al.*, 2000 ▸; Daniels *et al.*, 2004 ▸; Duguid *et al.*, 2005 ▸), *Pyrococcus kodakaraensis* (41%; Hashimoto *et al.*, 1999 ▸), *E. coli* (31%; Moore *et al.*, 1994 ▸), *Mycobacterium tuberculosis* (35%; Miggiano *et al.*, 2013 ▸, 2016 ▸) and *Saccharolobus solfataricus* (68%; Perugino *et al.*, 2015 ▸; Morrone *et al.*, 2017 ▸; Rossi *et al.*, 2018 ▸). The CTDs of the MGMTs are highly similar across species, whereas the NTDs are quite variable (Fig. 1 ▸). The NTD of StoMGMT was composed of an antiparallel β-sheet consisting of three β-strands and one interconnected folded helix (h1–H3, consisting of two 3_10_-helices and one α-helix). The β-sheet was connected to h1 through a loop between Asp27 of β3 and Glu33 of h1. Within this loop region, there were two cysteine residues, Cys29 and Cys31, which formed a disulfide bond and showed two different conformations in all of the crystals except for the Y91F/C120S-*O* ^6^-mdG crystal (Fig. 1 ▸). This disulfide bond, which is also present in *S. solfataricus* MGMT, is a characteristic feature of thermophilic proteins and is necessary for thermal stability (Perugino *et al.*, 2015 ▸). Interestingly, varied conformations of this disulfide bond of MGMT are exclusive to *S. tokodaii*, although the exact reason for this is obscure. Even the most similar ortholog of StoMGMT, *S. solfataricus* MGMT, does not show such multiple conformations of the disulfide bond. The H3 helix exposed to the solvent was located along the β1 strand; the CTD was connected to the NTD through a connecting loop consisting of the region between Lys56 and Phe69. The helix of H5 and H6 consists of a HTH motif, which binds to the minor groove of DNA. The short H7 helix in the middle of the CTD contained the catalytic Cys120 residue in the conserved PCHRV motif. The last H9 helix of the CTD was not visible in any structure except for the Y91F and C120S mutants.

**Figure 2:**
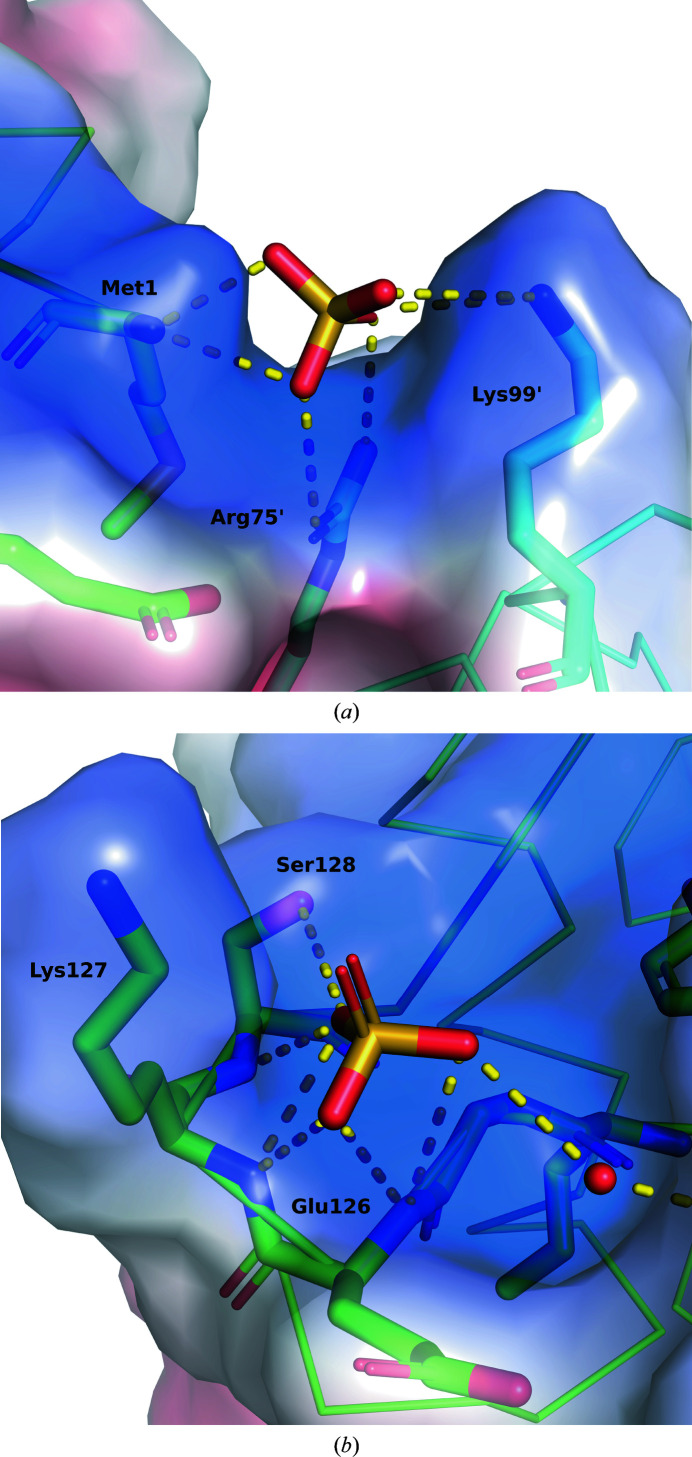
Electrostatic surfaces of Wild binding to two sulfate ions, which are bound to the NTD (*a*) and CTD (*b*). The positively charged surface is colored blue while the negatively charged surface is colored red.

One or two sulfate ions were found in all structures except for the Y91F/C120S–*O^6^*-mdG structure. The first was found in a positively charged saddle consisting of the N atom of Met1 and the side chains of Arg75 and Lys99 from the neighboring molecule (Fig. 2 ▸ *a*). The two basic amino acids are thought to be side chains involved in DNA binding based on comparisons with MGMTs from other species (Perugino *et al.*, 2015 ▸). The sulfate ion that connects the two molecules may be an artifact that maintains the crystal structure. The second, found only in the wild type (Wild), was bound to the N atoms of the main chains of Glu126 and Lys127 through hydrogen bonds (Fig. 2 ▸ *b*). This part is adjacent to the active pocket of the substrate and mimics the position of the phosphate group in the DNA.

**Figure 3:**
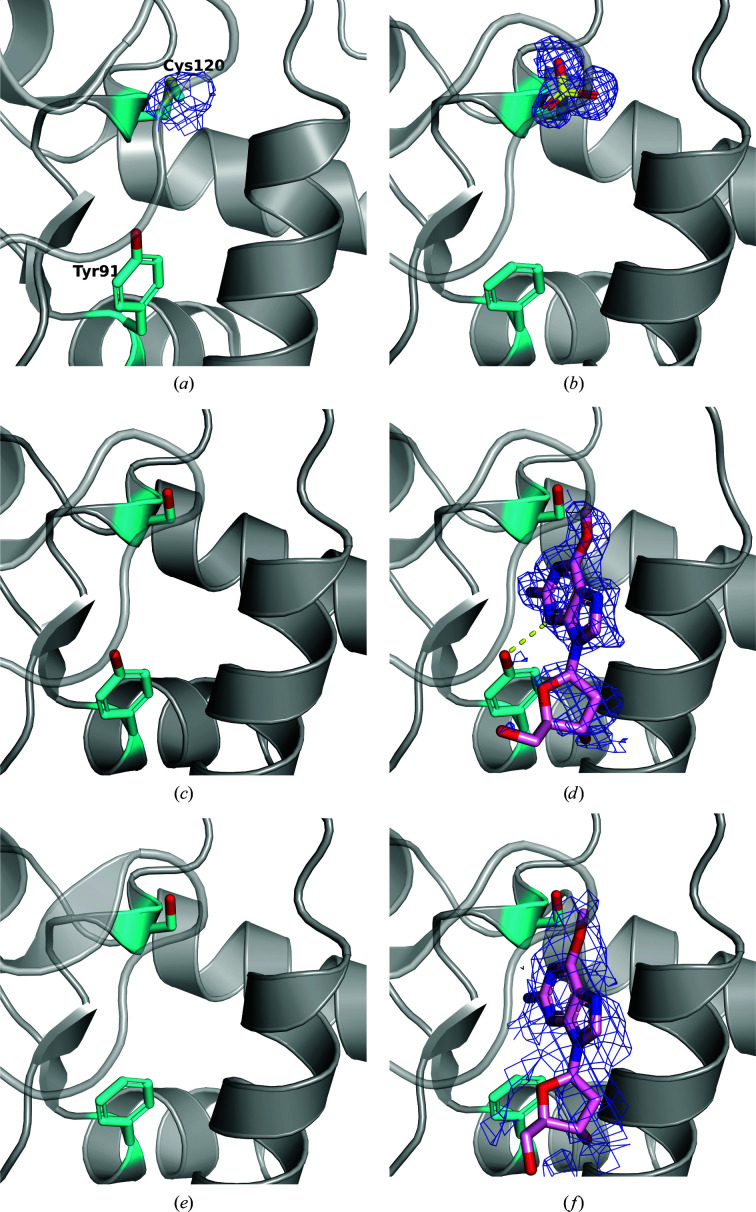
Close-up of active-site cleavage in StoMGMTs. (*a*) Wild^m^, (*b*) Y91F, (*c*) C120S, (*d*) C120S–*O* ^6^-mdG, (*e*) Y91F/C120S and (*f*) Y91F/C120S–*O* ^6^-mdG. The loop region between β5 and H8 is shown as a transparent structure. C, O and S atoms in the side chains of Tyr91 (Phe91) and Cys120 (Ser120) are colored cyan, red and yellow, respectively. Omit *F*_o_ − *F*_c_ difference maps (blue mesh), calculated without the methyl group (*a*), sulfo group (*b*), *O* ^6^-mdG (*d*) and *O* ^6^-mdG (*f*), are contoured at 2σ, 2σ, 2σ and 1σ, respectively.

The structures of the mutant crystals were as expected: in the Y91F mutant electron density for the hydroxy group of Tyr91 was not observed (Fig. 3 ▸ *b*), in the C120S mutant the S atom of Cys120 was replaced by an O atom, resulting in serine (Fig. 3 ▸ *c*), and in the Y91F/C120S mutant the two mutations occurred simultaneously (Fig. 3 ▸ *e*). The root-mean-square displacements (r.m.s.d.s) between the individual structures of Wild and the new mutants averaged 0.24 (±0.14) Å for all main-chain atoms and 0.93 (±0.26) Å for all protein atoms. These crystal structures showed almost no structural changes except in the mutated part.

The Wild^m^, C120S–*O^6^*-mdG and Y91F/C120S–*O^6^*-mdG structures were obtained and analyzed using crystals obtained by soaking the proteins in reservoir solution with *O* ^6^-mdG. The crystal structure of Wild^m^ had a methyl group attached to the S atom at the γ-position of the Cys120 residue in the active site (Fig. 3 ▸ *a*). However, electron density for *O* ^6^-mdG or 2′-deoxyguanine (dG) was not observed in this crystal structure. In contrast, in the crystal structure of C120S–*O* ^6^-mdG, electron density for *O* ^6^-mdG was obtained without the methyl group being transferred to the protein (Fig. 3 ▸ *d*). The N atom at position 3 of the base moiety of *O* ^6^-mdG and the hydroxy group of Tyr91 were hydrogen-bonded. The crystal structure of Y91F/C120S–*O* ^6^-mdG also showed electron density for *O^6^*-mdG, where the methyl group was not transferred to the protein (Fig. 3 ▸ *f*). However, electron density for *O* ^6^-mdG or dG was not found in the Y91F mutant crystal structure, regardless of whether it was soaked in *O* ^6^-mdG solution.

The position of the guanine base moiety in the crystal structures of C120S–*O* ^6^-mdG and Y91F/C120S–*O* ^6^-mdG appears to be the same as that of Cys119 and substrate DNA (Perugino *et al.*, 2015 ▸) in MGMT from *S. solfataricus*. In both structures, the r.m.s.d. of the superposition of this amino acid and the guanine moiety was approximately 0.18 Å. This means that the *O* ^6^-mdG in this study can be regarded as having reproduced the position of the substrate. We initially thought that the hydroxy group of Tyr91 was necessary for positioning the methylated base portion of DNA in the active site. In the crystal structure of C120S–*O* ^6^-mdG, a hydrogen bond between the hydroxy group of Tyr91 and N3 of the purine base was observed. In the crystal structure of Y91F/C120S–*O* ^6^-mdG, electron density for bound *O* ^6^-mdG is ambiguous and the *B* factor of the *O* ^6^-mdG is high. Furthermore, the resolution is lower than that of other structures. Therefore, the hydroxy group of Tyr91 might contribute to stabilization of the binding of substrate. However, the crystal structure of Y91F/C120S–*O* ^6^-mdG suggests that the presence of the Tyr91 hydroxy group is not essential for the substrate base to be able to enter the binding pocket. The lack of electron density in the Wild^m^ crystal structure for dGs with lost methyl groups implies that substrates with rearranged methyl groups are rapidly removed from the enzyme. A previous study reported that the hydroxy group of Tyr91 may stabilize the repaired guanine by reducing its negative charge (Daniels *et al.*, 2004 ▸). The S atom of cysteine is more nucleophilic than the O atom at position 6 of guanine, and the methyl group readily rearranges to cysteine by an S_N_2 reaction (Moore *et al.*, 1994 ▸). In the crystal structure of the Y91F mutant, the thiol group of Cys120 was converted to a sulfo group (Cys-SO_3_H) with three O atoms attached to it (Fig. 3 ▸ *b*), even though no additional manipulation was performed. This means that the cysteine in the expressed protein was oxidized by some substances. Thus, the enzyme would be inactive because oxidized cysteine would not be able to function as a nucleophile in the reaction. Because no electron density for dG or a methyl group was observed in the structure of Y91F mutant crystals immersed in *O* ^6^-mdG, and because it was not oxidized in Wild, we speculate that the hydroxy group of Tyr91 may have prevented the oxidant from entering the active site due to its size and charge. This suggests that the role of tyrosine, which is highly conserved at the N-terminus of the HTH motif across species, may be to protect the active site of MGMT, which is a suicide enzyme that can only work once. However, due to the critical role of the physiological context in determining whether or not a specific mutation in a protein impacts its function, definitive proof of the role of Tyr91 in these StoMGMT mutations and in protecting Cys120 from oxidation will only be possible with *in vivo* studies.

In conclusion, the crystal structures of the wild type and the Y91F, C120S and Y91F/C120S mutants of the MGMT enzyme derived from *S. tokodaii* revealed that the hydroxy group of tyrosine may play a protective role in MGMTs by preventing oxidants from entering their active sites. Overall, our results may provide a framework for directing future studies aimed at understanding the molecular mechanisms by which high levels of conserved amino acids play a role in ensuring the integrity of suicide enzymes, in addition to promoting enzyme activity.

## Supplementary Material

PDB reference: *O* ^6^-methylguanine-DNA methyltransferase, wild type, 7dkn

PDB reference: wild type with methylated Cys120, 7dqr

PDB reference: C120S mutant, 7csm

PDB reference: Y91F mutant, 7dqt

PDB reference: Y91F/C120S mutant, 7d4v

PDB reference: C120S mutant, complex with *O* ^6^-mdG, 7e1p

PDB reference: Y91F/C120S mutant, complex with *O* ^6^-mdG, 7dqq

## Figures and Tables

**Table 1:** Macromolecule-production information

Source organism	*Sulfurisphaera tokodaii* strain 7^T^
Expression vector	pET-11a
Expression host	*E. coli*
Complete amino-acid sequence of the construct produced†	MIVYGLYKSPFGPITVAKNEKGFVMLDFCDCAERSSLDNDYFTDFFYKLDLYFEGKKVDLTEPVDFKPFNEFRIRVFKEVMRIKWGEVRTYKQVADAVKTSPRAVGTALSKNNVLLIIPCHRVIGEKSLGGYSRGVELKRKLLELEGIDVAKFIEK

† The positions of the site-directed mutations are underlined.

**Table 2:** Crystallization

PDB code	7dqt	7dqq	7dkn, 7dqr, 7csm, 7e1p, 7d4v
Method	Hanging-drop vapor diffusion	Hanging-drop vapor diffusion	Hanging-drop vapor diffusion
Plate type	VDX plate	VDX plate	VDX plate
Temperature (K)	293	293	293
Protein concentration (mg ml^−1^)	10	10	10
Buffer composition of protein solution	50 m*M* potassium phosphate pH 6.5, 50 m*M* potassium chloride, 0.1 m*M* EDTA	50 m*M* potassium phosphate pH 6.5, 50 m*M* potassium chloride, 0.1 m*M* EDTA	50 m*M* potassium phosphate pH 6.5, 50 m*M* potassium chloride, 0.1 m*M* EDTA
Composition of reservoir solution	0.2 *M* potassium chloride, 0.05 *M* HEPES pH 7.5, 35%(*v*/*v*) pentaerythritol propoxylate (5/4 PO/OH)	5%(*v*/*v*) Tacsimate pH 7.0, 0.1 *M* HEPES pH 7.0, 10%(*w*/*v*) polyethylene glycol monomethyl ether 5000	0.2 *M* ammonium sulfate, 0.1 *M* Tris pH 8.5, 25%(*w*/*v*) polyethylene glycol 3350
Volume and ratio of drop	4 µl, 1:1	4 µl, 1:1	4 µl, 1:1
Volume of reservoir (µl)	700	700	700

**Table 3:** Data collection and processing Values in parentheses are for the outer shell.

	Wild	Wild^m^	Y91F	C120S	C120S–*O* ^6^-mdG	Y91F/C120S	Y91F/C120S–*O* ^6^-mdG
PDB code	7dkn	7dqr	7dqt	7csm	7e1p	7d4v	7dqq
Diffraction source	BL-1A, PF	BL-1A, PF	BL-5A, PF	BL-5A, PF	BL-5A, PF	BL-5A, PF	BL-5A, PF
Wavelength (Å)	1.1000	1.1000	1.0000	1.0000	1.0000	1.0000	1.0000
Temperature (K)	100	100	100	100	100	100	100
Detector	EIGER X 4M	EIGER X 4M	PILATUS3 2M	PILATUS3 6M	PILATUS3 6M	PILATUS3 6M	PILATUS3 6M
Crystal-to-detector distance (mm)	125.2	92.3	109.4	183.0	287.1	336.0	336.0
Rotation range per image (°)	0.1	0.1	1.0	0.1	0.1	0.1	0.1
Total rotation range (°)	180	180	360	180	180	180	180
Exposure time per image (s)	0.2	0.2	5.0	0.5	0.1	0.5	0.5
Space group	*P*2_1_2_1_2_1_	*P*2_1_2_1_2_1_	*P*2_1_2_1_2_1_	*P*2_1_2_1_2_1_	*P*2_1_2_1_2_1_	*P*2_1_2_1_2_1_	*P*2_1_2_1_2_1_
*a*, *b*, *c* (Å)	48.07, 52.71, 61.62	48.21, 51.47, 61.78	48.10, 52.84, 61.84	48.21, 52.87, 62.09	48.33, 52.69, 61.65	47.85, 52.06, 62.34	48.47, 53.17, 62.09
α, β, γ (°)	90, 90, 90	90, 90, 90	90, 90, 90	90, 90, 90	90, 90, 90	90, 90, 90	90, 90, 90
Mosaicity (°)	0.195	0.260	0.161	0.072	0.098	0.092	0.189
Resolution range (Å)	40.05–1.79 (1.83–1.79)	48.21–1.74 (1.77–1.74)	40.17–1.13 (1.15–1.13)	48.41–1.25 (1.27–1.25)	48.33–1.63 (1.66–1.63)	47.85–1.78 (1.82–1.78)	48.47–2.60 (2.72–2.60)
Total No. of reflections	93147 (2696)	107753 (6393)	755764 (33246)	285375 (13707)	129783 (6645)	98283 (5625)	33206 (4225)
No. of unique reflections	15203 (815)	16659 (926)	59665 (2845)	44164 (2124)	20281 (1003)	15471 (867)	5304 (642)
Completeness (%)	99.3 (92.4)	99.9 (100.0)	99.9 (97.7)	99.1 (97.9)	100.0 (100.0)	99.9 (100.0)	100.0 (100.0)
Multiplicity	5.9 (3.3)	6.5 (6.9)	12.7 (11.7)	6.5 (6.5)	6.4 (6.6)	6.4 (6.5)	6.3 (6.6)
〈*I*/σ(*I*)〉	20.8 (6.2)	34.8 (11.3)	26.1 (11.9)	27.0 (9.3)	40.0 (9.7)	33.3 (9.5)	32.3 (12.9)
*R*_r.i.m._ †	0.060 (0.169)	0.034 (0.154)	0.069 (0.186)	0.041 (0.196)	0.027 (0.188)	0.034 (0.174)	0.044 (0.151)
Overall *B* factor from Wilson plot (Å^2^)	17.74	22.15	11.82	13.22	19.26	27.67	27.54

† Estimated *R*_r.i.m._ = *R*_merge_[*N*/(*N* − 1)]^1/2^, where *N* is the data multiplicity.

**Table 4:** Structure refinement Values in parentheses are for the outer shell.

	Wild	Wild^m^	Y91F	C120S	C120S–*O* ^6^-mdG	Y91F/C120S	Y91F/C120S–*O* ^6^-mdG
PDB code	7dkn	7dqr	7dqt	7csm	7e1p	7d4v	7dqq
Resolution range (Å)	40.05–1.79 (1.84–1.79)	39.99–1.74 (1.79–1.74)	30.94–1.13 (1.16–1.13)	38.11–1.25 (1.28–1.25)	30.86–1.63 (1.67–1.63)	39.96–1.78 (1.83–1.78)	38.24–2.60 (2.67–2.60)
Completeness (%)	99.1	99.9	99.8	98.9	99.9	99.8	99.9
No. of reflections, working set	15164 (976)	16613 (1136)	59597 (4032)	44110 (2999)	20232 (1378)	15427 (1044)	5274 (382)
No. of reflections, test set	726 (47)	893 (61)	2959 (221)	2184 (154)	949 (80)	656 (61)	265 (10)
Final *R*_cryst_	0.180 (0.180)	0.189 (0.190)	0.171 (0.190)	0.169 (0.184)	0.183 (0.194)	0.206 (0.217)	0.191 (0.240)
Final *R*_free_	0.219 (0.183)	0.226 (0.240)	0.197 (0.218)	0.192 (0.221)	0.223 (0.229)	0.245 (0.275)	0.284 (0.389)
Cruickshank DPI	0.1281	0.1194	0.0346	0.0444	0.0975	0.1336	—
No. of non-H atoms							
Protein	1207	1215	1235	1243	1221	1186	1195
Ion	10	5	5	5	5	5	0
Ligand	0	0	0	0	20	0	20
Water	106	100	208	229	145	55	41
Total	1323	1320	1448	1477	1391	1246	1256
R.m.s. deviations							
Bonds (Å)	0.011	0.013	0.018	0.018	0.013	0.012	0.008
Angles (°)	1.689	1.866	2.176	2.095	1.898	1.748	1.571
Average *B* factors (Å^2^)							
Protein†	18.2	22.9	11.7	13.2	19.6	29.8	29.0
Ion	55.0	39.4	15.9	20.5	43.8	67.9	0.0
Ligand	—	—	—	—	24.2	—	58.2
Water	25.3	29.8	23.3	23.7	29.2	33.0	24.5
Ramachandran plot							
Favored regions (%)	95.3	95.9	95.4	94.8	94.6	96.6	94.6
Additionally allowed (%)	4.0	3.4	3.9	4.5	4.7	2.7	4.7

† The average *B* factors include multiple conformations.
